# Introdutory Lecture, Delivered before the Class of Institutes of Medicine, in Jefferson Medical College

**Published:** 1846-09

**Authors:** 


*Introductory Lecture, delivered before the Class of Institutes of Medicine, in Jefferson Medical College, November* 3, 1845. By Robley Dungleson, M. D. Published by the Class.

We have seldom read an introductory which contained more practical good sense than the one before us. A considerable portion of it is taken up in discussing the importance of a knowledge of the Latin language to the medical practitioner, and while the learned professor does not underrate its value, he does not think a profound knowledge of it indispensable as a preparatory qualification. He points out some very palpable errors which many medical writers have committed in introducing into their works miscellaneous matter not absolutely necessary to be understood by the student or practitioner. He represents it, very properly, as a species of glorification in which there is a positive evil. In proof of which he cites several examples. Of these we will quote one.

"*Mutton Suet*—Sevurn of the Pharmacopœia of the United States—is an officinal article of the Materia Medica; and is described in that national work ("Drs. Thomson and Pereira's Materia Medica") "as the prepared suet of the *Ovis Aries*," the sheep. Dr. Pereira, however, deems it necessary to give all the zoological characters of the sheep, generic and specific, with the figures of two rams, borrowed from another work, as if to convey the idea, that to judge of good suet, a knowledge of the animal producing it is indispensable or advisable."

The lecture also contains much good advise, and we doubt not the class were profited by it. It contains facts which could hardly fail to benefit any one, and uttered as they are by a man of such extensive experience, and varied and profound learning, they carry with them a weight entitled to more than ordinary consideration.—*Balt. Ed.*

